# Effect of red blood cell transfusion on the development of retinopathy of prematurity: A systematic review and meta-analysis

**DOI:** 10.1371/journal.pone.0234266

**Published:** 2020-06-08

**Authors:** Zhe Zhu, Xin Hua, Yong Yu, Pan Zhu, Kairui Hong, Yefang Ke

**Affiliations:** 1 Department of Blood Transfusion, Hwa Mei Hospital, University of Chinese Academy of Sciences, Ningbo, Zhejiang, China; 2 Ningbo Institute life and Health Industry, University of Chinese Academy of Sciences, Ningbo, Zhejiang, China; 3 Department of Clinical Laboratory, Hwa Mei Hospital, University of Chinese Academy of Sciences, Ningbo, Zhejiang, China; 4 Neonatal intensive care unit, Ningbo Women & Children’s Hospital, Ningbo, Zhejiang, China; 5 Department of Clinical Laboratory, Ningbo Women & Children’s Hospital, Ningbo, Zhejiang, China; The Ohio State University, UNITED STATES

## Abstract

**Background:**

The effect of red blood cell (RBC) transfusion on retinopathy of prematurity (ROP) is difficult to establish, because ROP may also be influenced by other factors. Therefore, we carried out a systematic review and meta-analysis to explore the relationship between RBC transfusion and the development of ROP.

**Methods:**

The PubMed, Embase, Cochrane Library and Web of Science databases were searched from their inception to September 1, 2019. Observational studies that reported the relationship between RBC transfusion and ROP after adjusting for other potential risk factors were included. The combined result was analyzed by a random effect model. Heterogeneity and publication bias were tested, and sensitivity analysis was performed.

**Results:**

Of the 2628 identified records, 18 studies including 15072 preterm infants and 5620 cases of ROP were included. A random effect model was used and revealed that RBC transfusion was significantly associated with ROP (pooled OR = 1.50, 95% CI: 1.27–1.76), with moderate heterogeneity among the included studies (I^2^ = 44.2%). Subgroup analysis indicated that RBC transfusion was more closely related to ROP in the group with a gestational age (GA) ≤32 weeks (OR = 1.77, 95% CI: 1.29–2.43) but not in the groups with a GA ≤34 weeks (OR = 1.36, 95% CI: 0.85–2.18) or a GA <37 weeks (OR = 1.25, 95% CI: 0.86–1.82). No obvious publication bias was found based on the funnel plot and Egger’s test. Removing any single study did not significantly alter the combined result in the sensitivity analysis.

**Conclusions:**

Our study revealed that RBC transfusion is an independent risk factor for the development of ROP, especially in younger preterm infants. However, there seemed to be no evidence to support an effect of RBC transfusion on ROP in older groups. Further studies addressing this issue in older preterm neonates are warranted.

## Introduction

Retinopathy of prematurity (ROP) is a vasoproliferative disorder affecting the retinas of preterm infants and is the leading cause of childhood blindness worldwide [1]. ROP is affected by multiple factors, such as maternal, perinatal, infant and treatment factors, and among these factors, red blood cell (RBC) transfusion may play an important role [2].

Due to the immature hematopoietic system and iatrogenic phlebotomy losses, preterm infants frequently undergo transfusion [3]. Reports have found that approximately 90% of extremely low birth weight (ELBW) infants receive at least one RBC transfusion [3]. In general, RBC transfusion is able to improve anemia, increase tissue oxygenation, promote growth and reduce mortality [4, 5]. However, considerable evidence suggests that RBC transfusion is related to several preterm disorders, including the development of ROP [5]. According to a national survey by Ludwig [6], the incidence of ROP in the transfusion group was 1.68-times as high as that in the non-transfusion group. Other studies did not identify a close relationship between RBC transfusion and ROP, especially after adjusting for other risk factors [7, 8]. Moreover, the most severely ill infants receive more RBC transfusions [9], making it difficult to identify the effect of RBC transfusion on ROP.

Therefore, a systematic review and meta-analysis was carried out to investigate the effect of RBC transfusion on the development of ROP. Because other risk factors may make it difficult to identify the role of RBC transfusion in ROP, only original studies reporting a relationship between RBC transfusion and ROP after adjusting for other potential risk factors were included.

## Materials and methods

### Search strategy

This systematic review and meta-analysis was performed according to the Meta-analysis of Observational Studies in Epidemiology (MOOSE) and the Preferred Reporting Items for Systematic Reviews and Meta-Analysis (PRISMA) statement [10, 11]. The PubMed, Embase, Cochrane Library and Web of Science databases were searched from their inception to September 1, 2019. The search was performed using combinations of the following keywords: “preterm infants”, “red blood cell transfusion” and “retinopathy of prematurity” without language limitation. The detailed search strategy for PubMed is shown in S1 Table. Moreover, reference lists from the key articles were also searched manually.

### Inclusion and exclusion criteria

The inclusion criteria included the following: (1) Original studies that reported the adjusted odds ratio (OR) and 95% confidence interval (CI) between RBC transfusion (yes or no) and any stage of ROP after adjusting for other potential confounding risk factors and (2) observational studies, including case-control and cohort studies. Duplicate studies, review articles, case reports, letters to editors, conference abstracts and articles with no available data were excluded.

### Data extraction

Two reviewers (ZZ and XH) independently selected studies and extracted data using a form that included study design, area, publication year, sample size, the main inclusion criteria, exposures and outcomes. If disagreements occurred, they were resolved by the third reviewer (PZ).

### Quality assessment

The Newcastle-Ottawa Scale was selected to assess the quality of the included studies. This scale, with a maximum of 9 points, is made up of three parts: patient selection (4 points), comparability of the study groups (2 points) and exposure/outcome (3 points). A study with a score below 6 points was considered to be of low quality.

### Statistical analysis

This meta-analysis was performed using Stata 16.0 software (StataCorp LP, College Station, Texas). Adjusted ORs with 95% CIs from the included studies were extracted, and the combined result was analyzed by using a random effect model with the DerSimonian-Laird method and displayed on forest plots. Heterogeneity was assessed using the chi-square test, and I^2^ was calculated; I^2^ values of 25%, 50%, and 75% indicated low, moderate and high heterogeneity [12]. Predefined subgroup analyses were performed according to study design, sample size, GA area, and year of publication. Moreover, we used funnel plots and Egger’s test to examine publication bias and utilized sensitivity analysis to assess the stability of the combined result.

## Results

### Search results and characteristics of eligible studies

**Fig 1:**
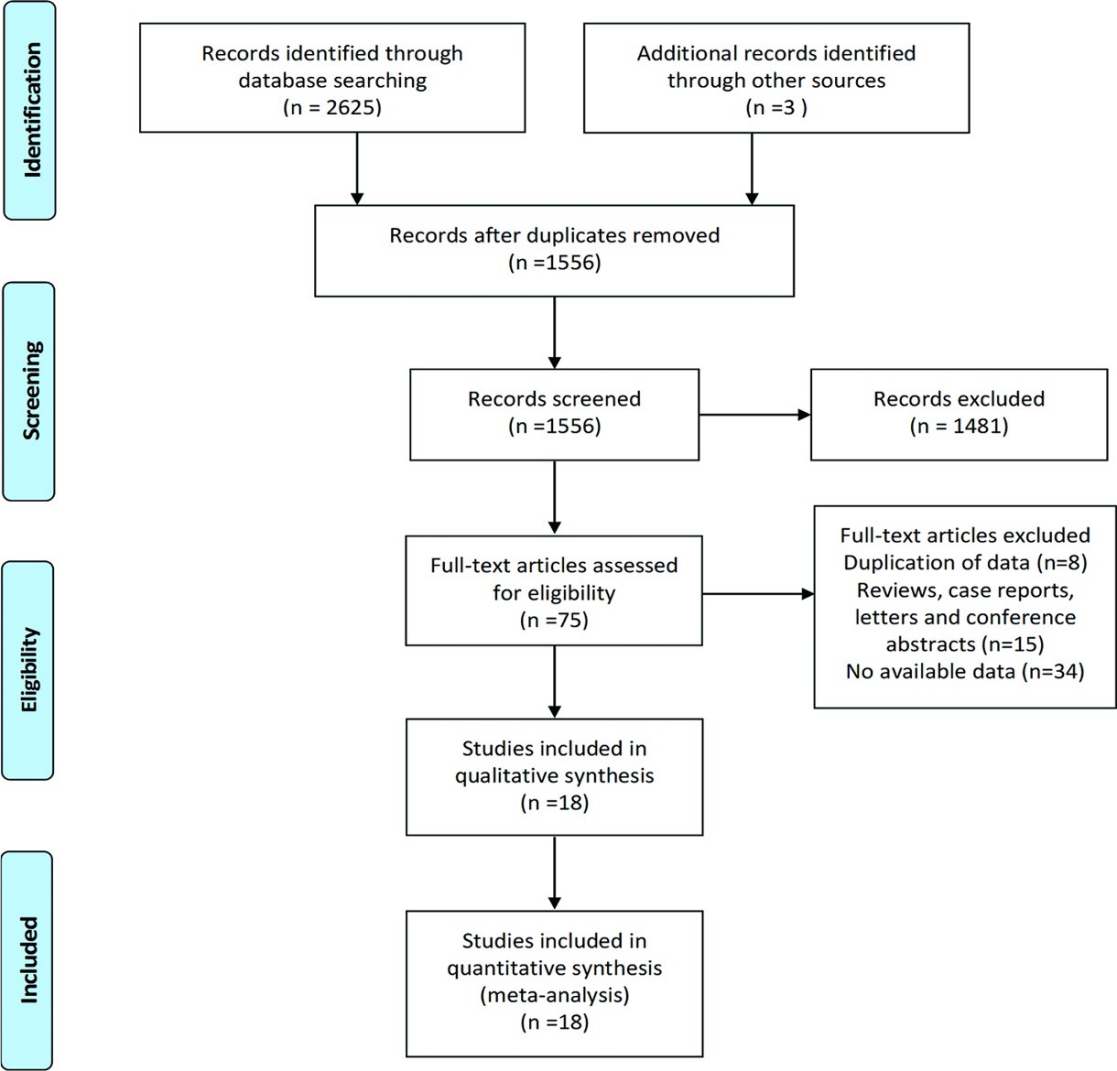
PRISMA flow diagram of studies selection.

A total of 2628 records were retrieved from the electronic and manual search. After duplicate removal, abstract screening and full-text article review, 18 studies with 15072 preterm infants and 5620 cases of ROP were included in this meta-analysis (Fig 1). The characteristics of the eligible studies are shown in Table 1. The NOS scores of the included studies ranged from 6 to 8, and detailed information is shown in S2 Table.

**Table 1:** Characteristics of included studies.

Author	Year of publication	Sample size	ROP cases	Included criteria	Area	Study design	Adjusted OR (95% CI)	Adjustments	NOS score
Zarei [13]	2019	1990	575	GA <37 weeks	Iran	cohort	1.355 (1.001–1.833)	GA, BW, gender, multiple gestation, oxygen therapy, IVP, sepsis, RDS, phototherapy, Intubation	7
Akkawi [7]	2019	115	27	BW <1500 g, GA ≤32 weeks	Palestine	case- control	2.642 (0.66–10.567)	GA, BW, multiple gestation, sibling affected by ROP, Hb at birth, RDS, lowest Hb, maximum bilirubin, days on mechanical ventilation, days on non-mechanical ventilation, days on oxygen therapy	6
Wu [14]	2018	504	131	BW <1500 g	China	cohort	1.819 (1.043–3.163)	maternal age, singleton gestation, IVF, preeclampsia, ICP, GA <32weeks, BW <1000g, gender, apnea, RDS, BPD, sepsis, PDA, hyperglycemia, surfactant use, dexamethasone use, IMV	8
Sathar [15]	2018	812	203	BW ≤1500 g, GA ≤32 weeks	India	cohort	2.567 (1.456–4.528)	BW <1500g, GA, pregnancy induced hypertension, premature rupture of membranes, intrauterine growth retardation, congenital pneumonia, apnea, sepsis, NEC, shock, birth asphyxia, hyaline membrane disease, surfactant, continuous positive airway pressure, phototherapy, oxygen therapy >7days, ventilator	7
Alshaikh [16]	2017	282	76	BW <1500g, GA <31 weeks	Canada	cohort	1.567 (1.198–2.04)	GA, BW, SNAP-PE score, oxygen therapy days, ventilator days, caesarean section, Caucasian, chorioamnionitis, gender, IUGR, RDS, PDA, surfactant use, sepsis, IVH, NEC, BPD	6
Yau [8]	2016	513	95	BW ≤1500 g, GA ≤32 weeks	Hong Kong	case- control	1.28 (0.18–13.17)	GA, BW, preeclampsia, gestational diabetes mellitus, IVF, postnatal hypotension, inotrope use, BPD, surfactant use, invasive mechanical ventilation, mean oxygen concentration, patent ductus arteriosus, NSAID use, anemia, IVH, hypoglycemia	7
Huang [17]	2015	5718	2785	BW <1500 g	Taiwan	cohort	1.26 (1.11–1.44)	preeclampsia, GA, BW, cesarean section, gender, GSA, Apgar score, RDS, PDA, sepsis	6
Ezz El Din [18]	2015	111	21	BW <1500 g, GA <32 weeks	Egypt	cohort	6.11 (1.22–30.44)	GA, BW, Gender, positive consanguinity, multiple gestation, vaginal delivery, maternal diabetes, maternal hypertension, PPROM, RDS, neonatal jaundice, IUGR, duration of admission, oxygen, ventilation, duration of ventilation, feeding, apnea, pneumothorax, BPD, PDA, inotropes, duration of inotropes, NEC, IVH, anemia, thrombocytopenia, sepsis, candida sepsis	7
Rao [19]	2013	282	61	BW <1500 g, GA ≤ 32 weeks	India	cohort	1.37 (0.48–3.96)	GA, BW, surfactant, sepsis, apnea, IPPV	7
Küçükevcilioǧlu [20]	2013	640	240	BW <1501 g, GA ≤34 week	Turkey	cohort	1.508 (0.892–2.552)	GA, BW, oxygen therapy, mechanical ventilation, RDS, sepsis, IVH	7
Isaza [21]	2013	423	171	BW <1500 g, GA <32 weeks	Canada	cohort	0.7 (0.36–1.36)	GA, BW, gender, days on ventilation therapy, perinatal infection, IVH, PDA, NEC	7
Akçakaya [22]	2012	517	177	GA <37 weeks	Turkey	case- control	0.651 (0.333–1.274)	GA, BW, sepsis, oxygen therapy, RDS, mechanical ventilation	7
Fortes [23]	2011	324	97	BW ≤1500 g, GA ≤32 weeks	Brazil	cohort	2.901 (1.533–5.49)	GA, maternal preeclampsia, antenatal steroid treatment, essential hypertension, IVH, use of oxygen in mechanical ventilation, use of indomethacin, vaginal delivery, SGA	7
Zhu [24]	2011	752	123	BW ≤2000 g	China	cohort	1.763 (1.034–3.008)	BW, asphyxia, apnea, oxygen therapy >5 days, RDS	8
Figueras-Aloy [25]	2010	718	225	BW ≤1500 g, GA ≤32 weeks	Spain	case-control	1.67 (1.04–2.66)	BW, cesarean, administrate of EPO+ Fe	7
Pinheiro [26]	2009	663	414	BW ≤1500 g, GA ≤36 weeks	Brazil	cohort	2.06 (1.11–3.83)	GA, duration of oxygen therapy	7
Mutlu [27]	2008	318	118	GA ≤34 weeks	Turkey	cohort	0.9 (0.31–2.58)	GA, BW, multiple births, oxygen therapy, mechanical ventilation, sepsis	7
Kim [28]	2004	390	81	GA <37 weeks	Korea	case- control	1.21 (0.7–2.1)	GA, BW, apnea, ventilator, surfactant, sepsis	7

GA: gestational age, BW: birth weight, IVH: intra ventricular hemorrhage, IVF: in vitro fertilization, ICP: intrahepatic cholestasis of pregnancy, IMV: invasive mechanical ventilation, SNAP-PE: Score for Neonatal Acute Physiology-Physiological Extension, IUGR: intrauterine growth restriction, PDA: patent ductus arteriosus, NSAID: nonsteroidal anti-inflammatory agent, SGA: small for gestational age, PPROM: preterm premature rupture of membranes, IPPV: invasive positive pressure ventilation, RDS: respiratory distress syndrome, EPO: erythropoietin, Fe: ferrum.

### Effects of RBC transfusion on ROP

**Fig 2:**
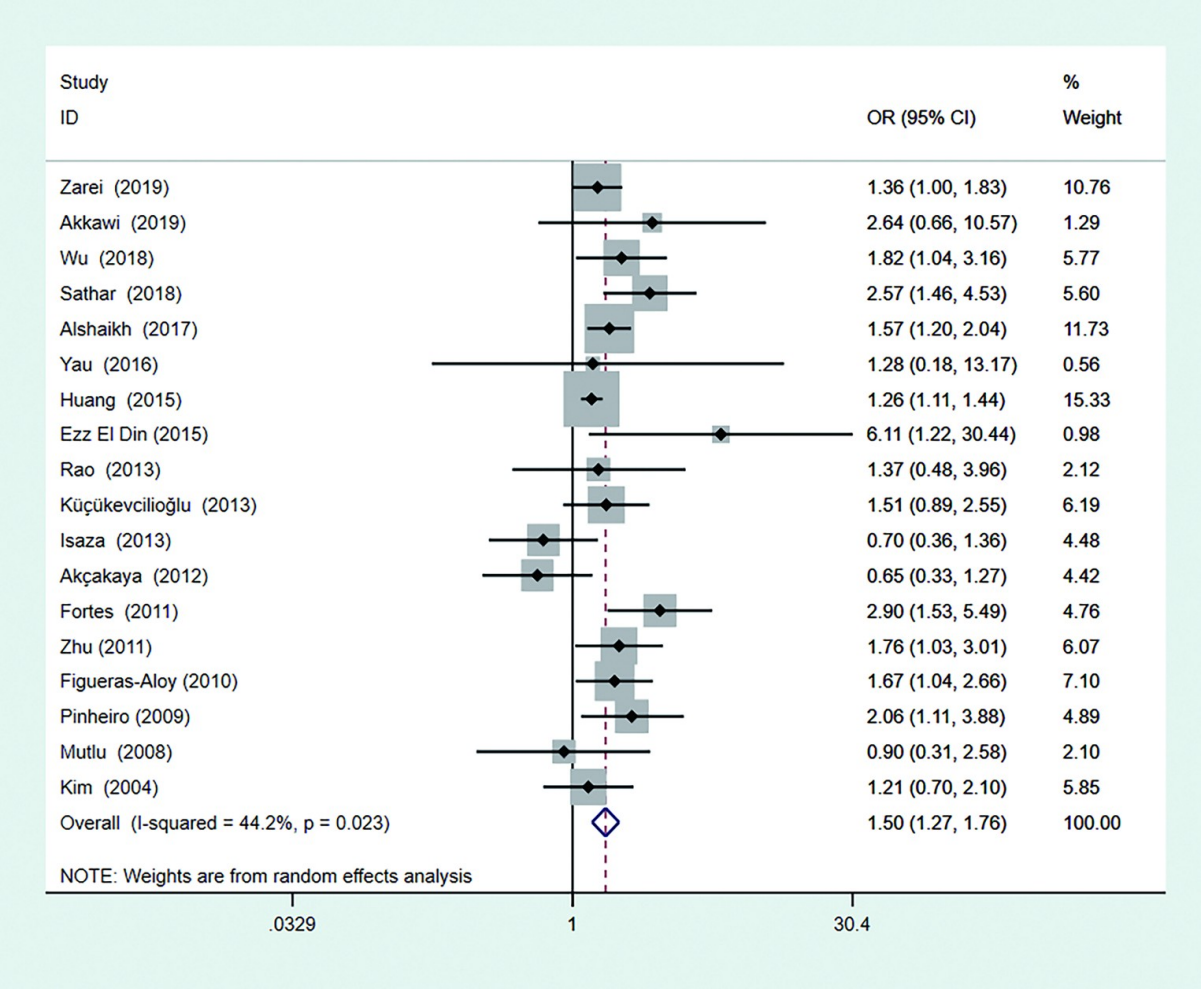
Forest plot for the association between RBC transfusion and the development of ROP. RBC: red blood cell, ROP: Retinopathy of prematurity.

Fig 2 shows the pooled results of 18 studies assessed using a random effect model, which indicated that RBC transfusion had a close relationship to ROP (pooled OR = 1.50, 95% CI: 1.27–1.76), with moderate heterogeneity among the included studies (I^2^ = 44.2%, 95% CI: 2.7%-67.9%).

### Subgroup analysis

**Fig 3:**
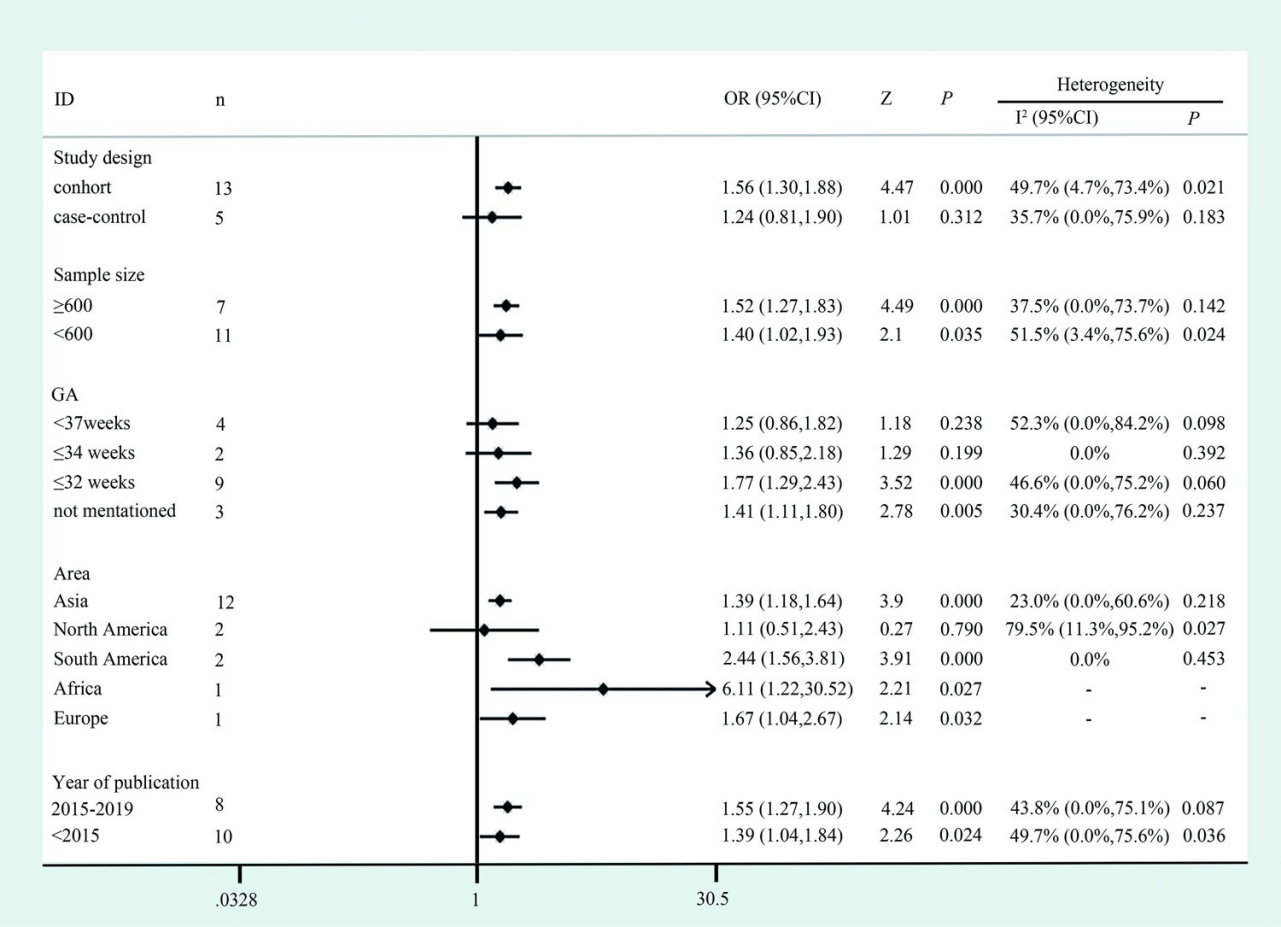
Subgroup analyses according to different study design, sample size, GA area and year of publication. GA: gestational age.

High heterogeneity was shown in studies conducted in the North American area (*P =* 0.027, I^2^ = 79.5%). Elevated risks were identified in studies with a cohort design, with a sample size ≥600, with a GA ≤32 weeks, conducted in South America and from published 2015–2019, with ORs of 1.56 (95% CI: 1.30–1.88), 1.52 (95% CI: 1.27–1.83), 1.77 (95% CI: 1.29–2.43), 2.44 (95% CI: 1.56–3.81) and 1.55 (95% CI: 1.27–1.90), respectively. However, there seemed to be no strong evidence to support an effect of RBC transfusion on ROP in case-control studies, studies performed in North America or studies that screened infants with a GA≤34 weeks or <37 weeks, with ORs of 1.24 (95% CI: 0.81–1.90), 1.11 (95% CI: 0.51–2.43), 1.36 (95% CI: 0.85–2.18), and 1.25 (95% CI: 0.86–1.82), respectively (Fig 3).

### Publication bias and sensitivity analysis

**Fig 4:**
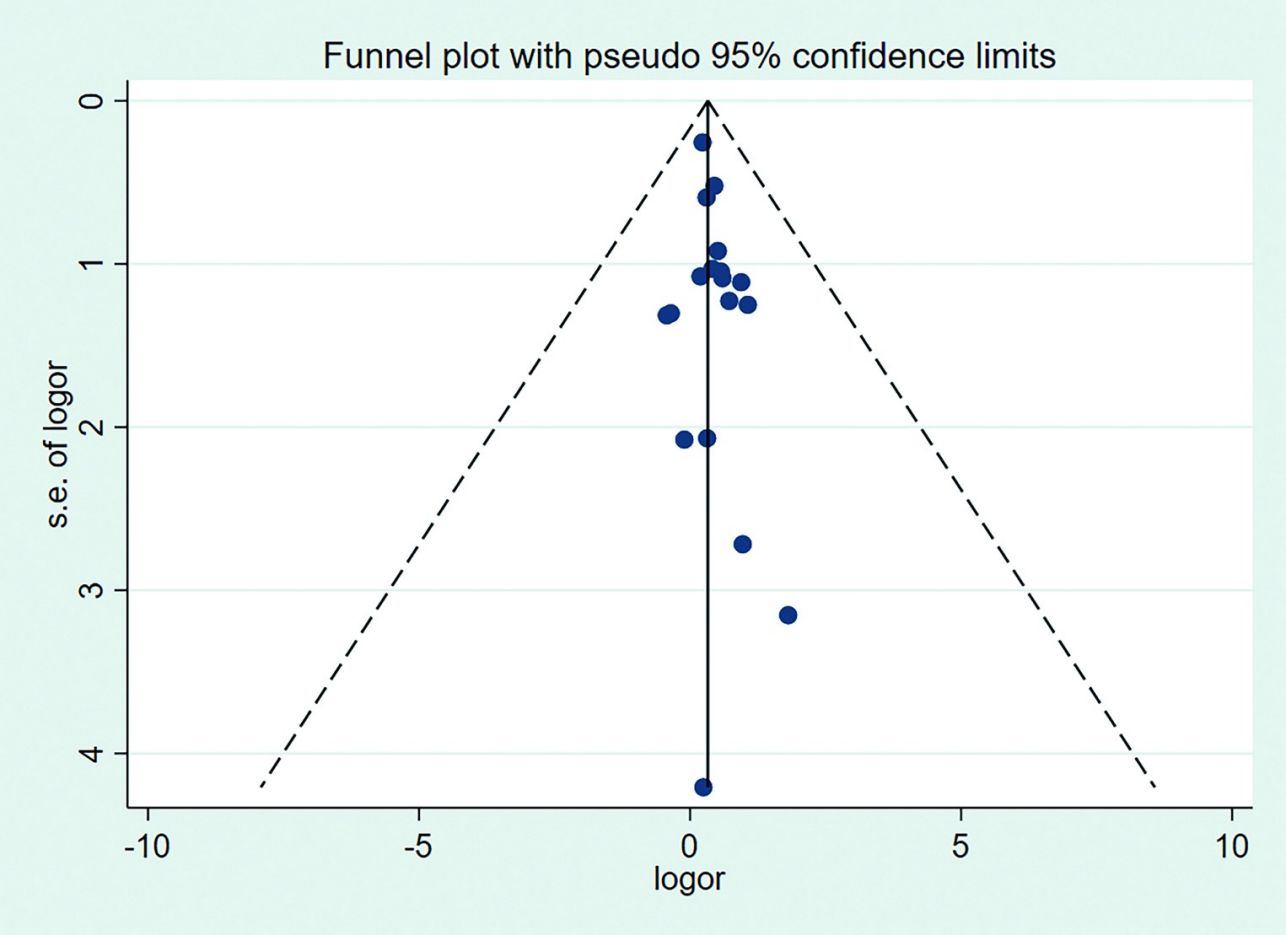
Funnel plot for the association between RBC transfusion and the development of ROP. RBC: red blood cell, ROP: Retinopathy of prematurity, or: odd ratio, s.e.: standard error.

Fig 4 shows the funnel plot of the studies included in this meta-analysis, and Egger’s test revealed a value of 1.11 (*P* = 0.287), which implied no obvious publication bias.

No apparent change was found in the pooled OR when any single study was removed, with a range from 1.44 (95% CI: 1.23–1.68) to 1.54 (95% CI: 1.28–1.86).

## Discussion

In this systematic review and meta-analysis, 13 cohort and 5 case-control studies including 15072 preterm infants and 5620 cases of ROP were included; among these studies, 10 indicated that RBC transfusion was significantly associated with ROP after adjusting for other confounding factors [13–18, 23–26], while others did not demonstrate this association [7, 8, 19–22, 27, 28]. Combining all of these studies, we found that RBC transfusion played a driving role in the development of ROP in preterm infants, with a pooled OR of 1.50 (95% CI: 1.27–1.76). Moderate heterogeneity was shown among the included studies (*P =* 0.023, I^2^ = 44.2%). No apparent publication bias was found according to the evaluation of the funnel plot and Egger’s test.

A subgroup analysis of case-control studies showed that there was no significant relationship between RBC and ROP (OR = 1.24, 95% CI: 0.81–1.90). The inclusion of few studies and the small sample size (5 studies including 2253 preterm infants and 650 ROP cases) may partially explain this. Another reason may relate to the included criteria, as 2 of the 5 case-control studies screened preterm infants with GA <37 weeks [29, 30]. This was confirmed in another subgroup study performed by GA, which showed that the risk of ROP attributed to RBC transfusion increases as GA decreases, with ORs of 1.77 (95% CI: 1.29–2.43) in the GA ≤32 weeks group, 1.36 (95% CI: 0.85–2.18) in the GA ≤34 weeks group and 1.25 (95% CI: 0.86–1.82) in GA <37 weeks group. There seemed to be no evidence to support an effect of RBC transfusion on ROP, which included older preterm infants in this meta-analysis. Previous studies have reported that the younger the preterm newborn is, the more frequently he or she requires transfused blood products [4, 31], and RBC transfusion times were associated with an increased risk of ROP [32, 33]. This phenomenon may suggest that the immature retina of younger infants is more susceptible to RBC transfusion effects.

ROP is a multifactorial disease, and one of the key elements in its development is oxidative damage [1], which is exactly what RBC transfusion results in. RBC transfusion influences ROP mainly in two ways. First, RBC transfusion increases iron intake, thereby increasing the level of its oxidation product. As early as 1997, Inder et al [34] discovered a close relationship between RBC transfusion, excessive iron load and ROP. Hirano’s study, in favor of such a view, further noticed that the same situation did not appear in full-term infants [35]. Second, unlike fetal hemoglobin (HbF), adult hemoglobin (HbA) has a lower affinity for oxygen, thereby shifting the oxygen-hemoglobin dissociation curve to the right and unloading more oxygen to the developing retina after the transfusion of adult blood products [5]. In a pilot prospective cohort study, Stutchfield et al [36] demonstrated that lower %HbF caused by transfusion is an independent risk factor for ROP. Additionally, biologically active substances in blood products may also play a role [5].

RBC transfusion is a life-saving therapeutic method in some emergency conditions, but it is known to inhibit the immune response and transmit infectious diseases [4]. Our findings again call upon doctors to weigh the risks of RBC transfusions against the benefits. Neonatologists have adopted several methods to reduce RBC transfusion in preterm infants. A systematic review and meta-analysis [37] demonstrated that the use of restrictive hemoglobin thresholds would reduce transfusion but had no significant impact on death or major morbidities. Other strategies include delayed cord clamping [38], milking of the umbilical cord [39] and autologous or allogenic umbilical cord blood transfusion [40, 41].

The strength of this meta-analysis was that it combined 18 observational studies that reported the effect of RBC transfusion on ROP after adjusting for other confounding risk factors. Limitations included the potential influence of different confounders and heterogeneity among the included studies. First, the confounders varied between different studies; some studies performed fully adjusted analyses, while others performed partially adjusted analyses. Moreover, maternal factors such as preeclampsia [42] and maternal diabetes [43] were seldom included as potential confounders in these studies. Second, moderate heterogeneity was found among the included studies. Third, although no obvious publication bias was found by funnel plot and Egger’s test, the number of studies included in this meta-analysis was still insufficient to make a certain assessment about the publication bias. These limitations must be considered when interpreting the final results.

## Conclusions

In conclusion, this systematic review and meta-analysis reveals that RBC transfusion is an independent risk factor for the development of ROP, especially in younger preterm infants. This calls upon doctors to weigh the risks of RBC transfusion against the benefits and avoid unnecessary transfusion in preterm infants. However, there seemed to be no evidence to support an effect of RBC transfusion on ROP in older groups. Further studies addressing this issue in older preterm neonates are warranted.

## Supporting information

S1 Table PubMed search strategy. (DOCX)

S2 Table The Newcastle-Ottawa Scale of included studies. (DOCX)

S1 Checklist PRISMA checklist. (DOC)
