# Supplementary material for: Effect of red blood cell transfusion on the development of retinopathy of prematurity: A systematic review and meta-analysis
Source: PLoS One. 2020 Jun 8;15(6):e0234266. doi: 10.1371/journal.pone.0234266 (PMC7279893; doi:10.1371/journal.pone.0234266)
Supplement: S1 Table — (DOCX) [file pone.0234266.s001.docx]

**S1 Table. PubMed search strategy.**

| 1.(“risk factor”[Title/Abstract]) OR (“risk factors”[Title/Abstract]) OR ("Blood Transfusion"[Mesh]) OR (“blood transfusion”[Title/Abstract]) OR (“blood transfusions”[Title/Abstract]) OR (“blood component transfusion”[Title/Abstract]) OR (“blood component transfusions”[Title/Abstract]) OR (“Erythrocyte Transfusion”[Title/Abstract]) OR (“Erythrocyte Transfusions”[Title/Abstract]) OR (“red blood cell transfusion”[Title/Abstract]) OR (“red blood cell transfusions”[Title/Abstract]) OR (“red cell transfusion”[Title/Abstract]) OR (“red cell transfusions”[Title/Abstract]) |
| --- |
| 2. (“low birth weight infant”[Title/Abstract]) OR (“low birth weight infants”[Title/Abstract]) OR(“low birth weight”[Title/Abstract]) OR (“low birth weights”[Title/Abstract]) OR(“very low birth weight infant”[Title/Abstract]) OR (“very low birth weight infants”[Title/Abstract]) OR (“very low birth weight”[Title/Abstract]) OR (“very low birth weights”[Title/Abstract]) OR (“Extremely Low Birth Weight infant”[Title/Abstract]) OR (“Extremely Low Birth Weight infants”[Title/Abstract]) OR (“Extremely Low Birth Weight”[Title/Abstract]) OR (“Extremely Low Birth Weight”[Title/Abstract])) OR ("Infant, Low Birth Weight"[Mesh]) |
| 3. (“premature infant”[Title/Abstract]) OR (“premature infants”[Title/Abstract]) OR (“preterm infant”[Title/Abstract]) OR(“preterm infants”[Title/Abstract]) OR (“extremely premature infant”[Title/Abstract]) OR (“extremely premature infants”[Title/Abstract]) OR (“extremely preterm infant”[Title/Abstract]) OR (“extremely preterm infants”[Title/Abstract]) OR ("Infant, Premature"[Mesh]) |
| 4. ("Retinopathy of Prematurity"[Title/Abstract]) OR (ROP[Title/Abstract]) OR (“Prematurity Retinopathy”[Title/Abstract]) OR (“Prematurity Retinopathies”[Title/Abstract]) OR (“Retrolental Fibroplasia”[Title/Abstract]) OR (“Retrolental Fibroplasias”[Title/Abstract]) OR ("Retinopathy of Prematurity"[Mesh]) |
| 5. 2 OR 3 |
| 6. 1 AND 5 AND 4 |
