# Supplementary material for: Effect of red blood cell transfusion on the development of retinopathy of prematurity: A systematic review and meta-analysis
Source: PLoS One. 2020 Jun 8;15(6):e0234266. doi: 10.1371/journal.pone.0234266 (PMC7279893; doi:10.1371/journal.pone.0234266)
Supplement: S2 Table — (DOCX) [file pone.0234266.s002.docx]

**S2 Table. The Newcastle-Ottawa Scale of included studies**

| Study | Patient selection | Comparability | Exposure/Outcome | NOS score |
| --- | --- | --- | --- | --- |
| Zarei 2019 | ★★☆★ | ★★ | ★☆★ | 7 |
| Akkawi 2019 | ★★☆★ | ★★ | ☆★☆ | 6 |
| Wu 2018 | ★★☆★ | ★★ | ★★★ | 8 |
| Sathar 2018 | ★★☆★ | ★★ | ★★☆ | 7 |
| Alshaikh 2017 | ★★☆★ | ★★ | ★☆☆ | 6 |
| Yau 2016 | ★★☆★ | ★★ | ★★☆ | 7 |
| Huang 2015 | ★★☆★ | ★★ | ★☆☆ | 6 |
| Ezz El Din 2015 | ★★☆★ | ★★ | ★★☆ | 7 |
| Rao 2013 | ★★★★ | ★★ | ★☆☆ | 7 |
| Küçükevcilioǧlu 2013 | ★★☆★ | ★★ | ★★☆ | 7 |
| Isaza 2013 | ★★☆★ | ★★ | ★★☆ | 7 |
| Akçakaya 2012 | ★★☆★ | ★★ | ★★☆ | 7 |
| Fortes 2011 | ★★☆★ | ★★ | ★★☆ | 7 |
| Zhu 2011 | ★★★★ | ★★ | ★★☆ | 8 |
| Figueras-Aloy 2010 | ★★☆★ | ★★ | ★★☆ | 7 |
| Pinheiro 2009 | ★★★★ | ★★ | ★☆☆ | 7 |
| Mutlu 2008 | ★★☆★ | ★★ | ★★☆ | 7 |
| Kim 2004 | ★★☆★ | ★★ | ★★☆ | 7 |
